# Septins restrict inflammation and protect zebrafish larvae from *Shigella* infection

**DOI:** 10.1371/journal.ppat.1006467

**Published:** 2017-06-26

**Authors:** Maria J. Mazon-Moya, Alexandra R. Willis, Vincenzo Torraca, Laurent Boucontet, Avinash R. Shenoy, Emma Colucci-Guyon, Serge Mostowy

**Affiliations:** 1 Section of Microbiology, MRC Centre for Molecular Bacteriology and Infection, Imperial College London, London, United Kingdom; 2 Institut Pasteur, Unité Macrophages et Développement de l'Immunité, Département de Biologie du Développement et des Cellules Souches, Paris, France; 3 CNRS, UMR 3738, Paris, France; University of Toronto, CANADA

## Abstract

*Shigella flexneri*, a Gram-negative enteroinvasive pathogen, causes inflammatory destruction of the human intestinal epithelium. Infection by *S*. *flexneri* has been well-studied *in vitro* and is a paradigm for bacterial interactions with the host immune system. Recent work has revealed that components of the cytoskeleton have important functions in innate immunity and inflammation control. Septins, highly conserved cytoskeletal proteins, have emerged as key players in innate immunity to bacterial infection, yet septin function *in vivo* is poorly understood. Here, we use *S*. *flexneri* infection of zebrafish (*Danio rerio*) larvae to study *in vivo* the role of septins in inflammation and infection control. We found that depletion of Sept15 or Sept7b, zebrafish orthologs of human SEPT7, significantly increased host susceptibility to bacterial infection. Live-cell imaging of Sept15-depleted larvae revealed increasing bacterial burdens and a failure of neutrophils to control infection. Strikingly, Sept15-depleted larvae present significantly increased activity of Caspase-1 and more cell death upon *S*. *flexneri* infection. Dampening of the inflammatory response with anakinra, an antagonist of interleukin-1 receptor (IL-1R), counteracts Sept15 deficiency *in vivo* by protecting zebrafish from hyper-inflammation and *S*. *flexneri* infection. These findings highlight a new role for septins in host defence against bacterial infection, and suggest that septin dysfunction may be an underlying factor in cases of hyper-inflammation.

## Author summary

*Shigella* are human-adapted *Escherichia coli* and cause bacillary dysentery via inflammatory destruction of the gut epithelium. In this study, we use a zebrafish (*Danio rerio*) model of *Shigella* infection to discover new roles for the cytoskeleton in inflammation and infection control. Septins, a poorly understood component of the cytoskeleton, are important in numerous biological processes including cell division and host-pathogen interactions. Here, we show that zebrafish septins can restrict inflammation and *Shigella* infection *in vivo*. In the absence of septins, larvae infected with *Shigella* exhibit increased mortality and bacterial burdens associated with increased Caspase-1 activity and neutrophil death. Pharmacological suppression of Il-1β signaling rescues septin-deficiency *in vivo* by reducing neutrophil death and preventing larval mortality. These findings reveal a new link between septins and inflammation, and highlight the cytoskeleton as a structural determinant of host defence.

## Introduction

Septins, a poorly understood component of the cytoskeleton, are highly-conserved guanosine triphosphate (GTP) binding proteins organized into 4 groups based on sequence homology (the SEPT2, SEPT3, SEPT6, and SEPT7 groups). Septins from different groups assemble into hetero-oligomeric complexes which can form non-polar filaments and ring-like structures [1]. By acting as scaffolds for protein recruitment and diffusion barriers for cellular compartmentalization, septins have key roles in numerous biological processes, including cell division and host-pathogen interactions [1, 2]. Studies using human epithelial cells have revealed important roles for septins in cell-autonomous immunity, showing that septins assemble into cage-like structures to prevent the dissemination of cytosolic bacteria polymerizing actin tails [3–5]. Septin cages have also been observed *in vivo* using bacterial infection of zebrafish (*Danio rerio*) larvae [6], yet roles for septins in innate immunity *in vivo* remain largely unexplored.

The inflammasome is an intracellular platform that assembles in response to infection to recruit and activate Caspase-1 [7]. Caspase-1 activation enables the processing and secretion of the proinflammatory cytokine interleukin 1β (IL-1β) to control infection. How the inflammasome is triggered and assembled is the subject of intense investigation [8, 9], and the mechanisms underlying inflammation regulation are poorly understood [5]. New work has shown that components of the cytoskeleton play important roles in innate immunity and are required for inflammation control [5]. Actin and other proteins involved in actin polymerization regulate the NLRP3 (NACHT, LRR and PYD domains-containing protein 3) inflammasome by interacting with Caspase-1 and other inflammasome components [10–12]. A separate study showed that actin depolymerization, as a consequence of mutations in WD repeat-containing protein (WDR1), can trigger disease by activation of the pyrin inflammasome [13, 14]. Other components of the cytoskeleton, including microtubules and the intermediate filament protein vimentin, promote NLRP3 activity by helping to recruit ASC (apoptosis-associated speck-like protein containing a caspase-recruitment domain) and stabilize NLRP3 inflammasomes, respectively [15, 16]. The role of the septin cytoskeleton in inflammation control has not yet been tested.

*Shigella*, a Gram-negative enteroinvasive pathogen, causes nearly 165 million illness episodes and over 1 million deaths annually [17]. Similar to other Gram-negative pathogens in hospital patients, cases of drug-resistant *Shigella* strains are rising [18]. To explore the innate immune response to *Shigella*, several infection models have been valuable, helping to discover key roles for NOD-like receptors (NLRs) [19], neutrophil extracellular traps (NETs) [20], bacterial autophagy [21], and inflammasomes [22] in host defence. Remarkably, the major pathogenic events that lead to shigellosis in humans (i.e., macrophage cell death, invasion and multiplication within epithelial cells, cell-to-cell spread, inflammatory destruction of the host epithelium), are faithfully reproduced in a zebrafish model of *S*. *flexneri* infection [6]. Exploiting the optical accessibility of zebrafish larvae, we now have the possibility to spatio-temporally examine the biogenesis, architecture, coordination, and resolution of the innate immune response to *S*. *flexneri in vivo*.

In this study, we use a *S*. *flexneri*-zebrafish infection model to discover new roles for septins in host defence. We show that zebrafish septins restrict inflammation and are required for neutrophil-mediated immunity. To rescue septin-deficiency *in vivo*, we used therapeutic inhibition of Il-1β signaling and prevent neutrophil death and larval mortality. These results demonstrate a previously unknown role for septins in inflammation and infection control, and highlight the cytoskeleton as a target for suppression of inflammation.

## Results

### Establishment of localized *S*. *flexneri* infection in the zebrafish hindbrain ventricle

**Fig 1:**
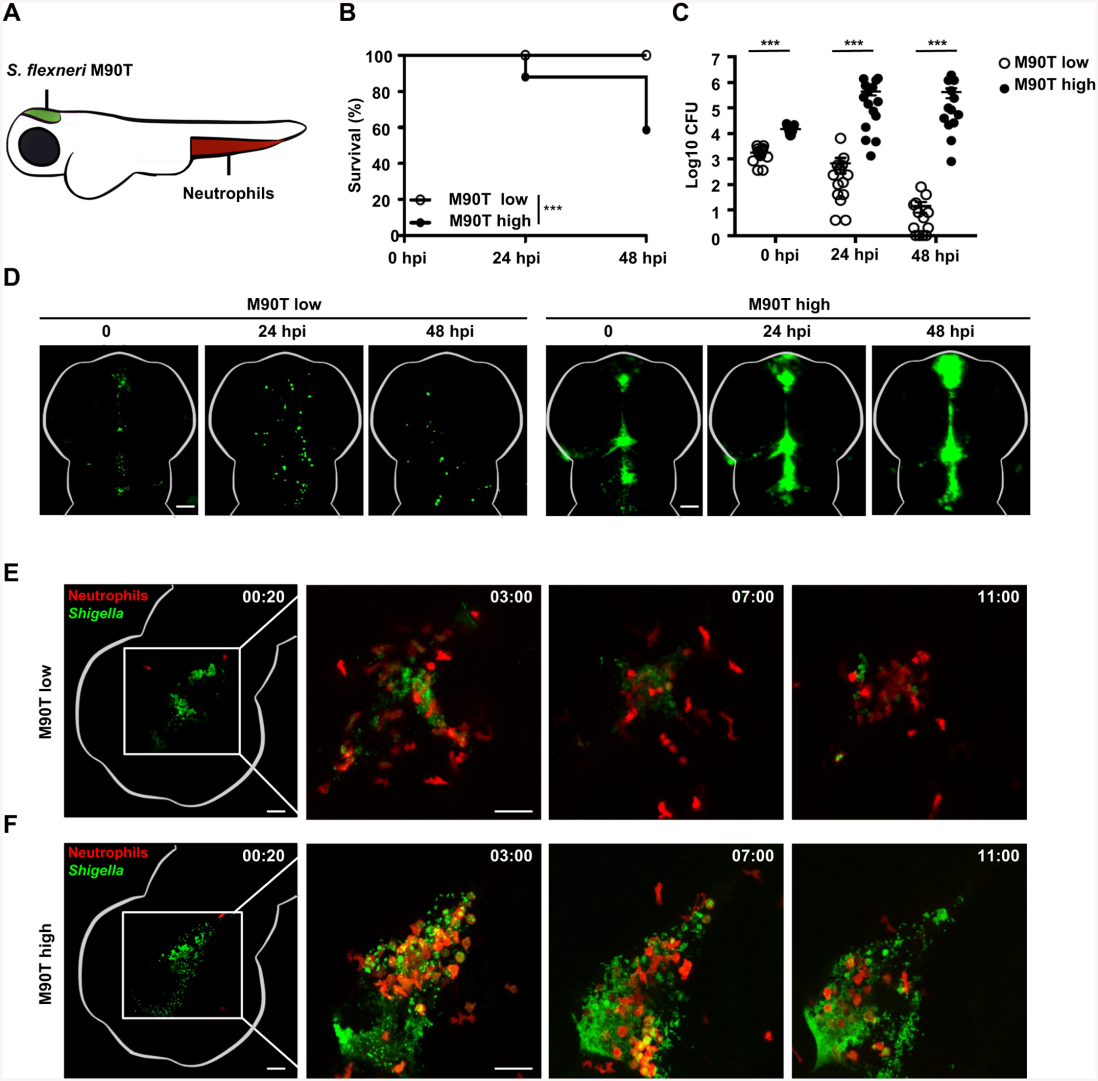
*S*. *flexneri* infection of the zebrafish hindbrain ventricle. A. Cartoon of zebrafish larva (3 dpf) showing localization of neutrophils prior to infection (red) and the site of *S*. *flexneri* M90T (green) injection in the HBV. B. Survival curves of larvae infected with a low (≤ 3 x 10^3^ CFU) or high (≥ 1 x 10^4^ CFU) dose of *S*. *flexneri* M90T. Pooled data from 5 independent experiments per inoculum class using at least 15 larvae per experiment. Significance testing performed by Log Rank test. ***, P<0.001. C. Enumeration of bacteria at 0, 24, or 48 hpi from surviving larvae infected with a low (open circles) or high (closed circles) dose of *S*. *flexneri* M90T. Pooled data from 5 independent experiments per inoculum class using up to 3 larvae per treatment. Circles represent individual larvae, and only larvae having survived the infection (thus far) included here (i.e., dead larvae not homogenised for counts). Mean ± SEM also shown (horizontal bars). Significance testing performed by Student’s t test. ***, P<0.001. D. Representative images of larvae infected in the HBV with low or high dose of GFP-*S*. *flexneri* M90T. For each dose, the same larva was imaged at 0, 24, and 48 hpi using a fluorescent stereomicroscope. Scale bars, 100 μm. E-F. Representative frames extracted from *in vivo* time-lapse confocal imaging of *lyz*:dsRed larvae (3 dpf, red neutrophils) injected in the HBV with a (E) low dose or (F) high dose of GFP-*S*. *flexneri* M90T. First frame 20 mpi, followed by frames at 3, 7, and 11 hpi. Maximum intensity Z-projection images (2 μm serial optical sections) are shown. Scale bars, 50 μm. See also S2 and S3 Videos.

The hindbrain ventricle (HBV) of zebrafish larvae is uniquely suited to analyze host-pathogen interactions because it is optically accessible and enables analysis of a directed leukocyte response to a compartmentalized infection (Fig 1A). We therefore characterized the HBV of zebrafish larvae as an infection site for *S*. *flexneri* M90T. Larvae aged 3 days post fertilization (dpf) were microinjected with a low (≤ 3 x 10^3^ CFU) or high (≥ 1 x 10^4^ CFU) dose of bacteria and their survival was assessed over time (Fig 1B). Larvae infected with a low dose of *S*. *flexneri* presented 100% survival, whereas a high dose of *S*. *flexneri* resulted in the death of ~40% of larvae within 48 hours post infection (hpi). We measured the bacterial load of infected zebrafish larvae over time by plating homogenates of viable larvae, excluding those that had already succumbed to infection. Larvae infected with a low dose of *S*. *flexneri* controlled bacterial replication, whereas larvae receiving a high dose of *S*. *flexneri* were associated with an increasing bacterial burden (Fig 1C).

To visualize the course of infection, larvae were infected with GFP-*S*. *flexneri* M90T and imaged by fluorescence microscopy. In agreement with bacterial enumerations, larvae that received a low dose inoculum showed limited proliferation of GFP-*S*. *flexneri* (Fig 1D). In contrast, larvae that received a high dose inoculum showed increasing bacterial burdens at 24 and 48 hpi (Fig 1D). Irrespective of the dose used, *S*. *flexneri* remained in the HBV and forebrain and did not cause systemic infection. Histological analyses of transverse sections of infected larvae confirmed the aggregation of *S*. *flexneri* on walls of the HBV (S1A Fig). In humans, *Shigella* infection and pathogenesis is strictly dependent upon the type III secretion system (T3SS) [23]. To test the role of the T3SS in our infection model, larvae were injected with T3SS-deficient (T3SS-) *S*. *flexneri* (Δ*mxiD* strain). The survival of zebrafish larvae infected with T3SS- *S*. *flexneri* at low or high dose was ~100% (S1B and S1C Fig), demonstrating that the T3SS contributes to *Shigella* virulence *in vivo*.

We next used the zebrafish HBV model of infection to study the control of *S*. *flexneri* by leukocytes. We outcrossed Tg(*mpeg1*:*Gal4-FF)*^*gl25*^*/*Tg(*UAS-E1b*:*nfsB*.mCherry)^*c264*^ (herein referred to as *mpeg1*:*G/U*:mCherry) with Tg(*mpx*:GFP)^*i114*^ (herein referred to as *mpx*:GFP) to generate double transgenic zebrafish larvae with red macrophages and green neutrophils. Larvae were infected with Crimson-*S*. *flexneri* M90T and leukocyte behavior recorded by confocal microscopy. Using a low dose of *S*. *flexneri*, we observed rapid aggregation of bacteria on walls of the HBV and by 12 hpi most bacteria had been cleared (S1D Fig, see also S1 Video). Here, macrophages were the first leukocytes to arrive (from 20 mpi) and engulf bacteria, however, as we have previously shown using caudal vein injections, *S*. *flexneri* were able to proliferate within macrophages and cause their death [6]. In contrast, neutrophils are massively recruited within hours and become the predominant leukocyte, and actively participate in the control of *S*. *flexneri* by engulfing both aggregates of extracellular bacteria and debris from macrophages unable to control infection. We thus infected Tg(*lyz*:dsRed)^*nz50*^ (herein referred to as *lyz*:dsRed) zebrafish embryos, a transgenic line in which dsRed is expressed specifically in neutrophils [24]. In the case of a low dose, neutrophils are recruited hours following infection and control *S*. *flexneri* proliferation (Fig 1E, see also S2 Video). In contrast, a high dose of *S*. *flexneri* results in uncontrolled bacterial proliferation and concomitant neutrophil cell death (Fig 1F, see also S3 Video). Collectively, these results demonstrate that infection of the zebrafish HBV is a valuable system to study *in vivo* the control of *Shigella* infection by neutrophils.

### Sept15 is required to control *S*. *flexneri* infection *in vivo*

**Fig 2:**
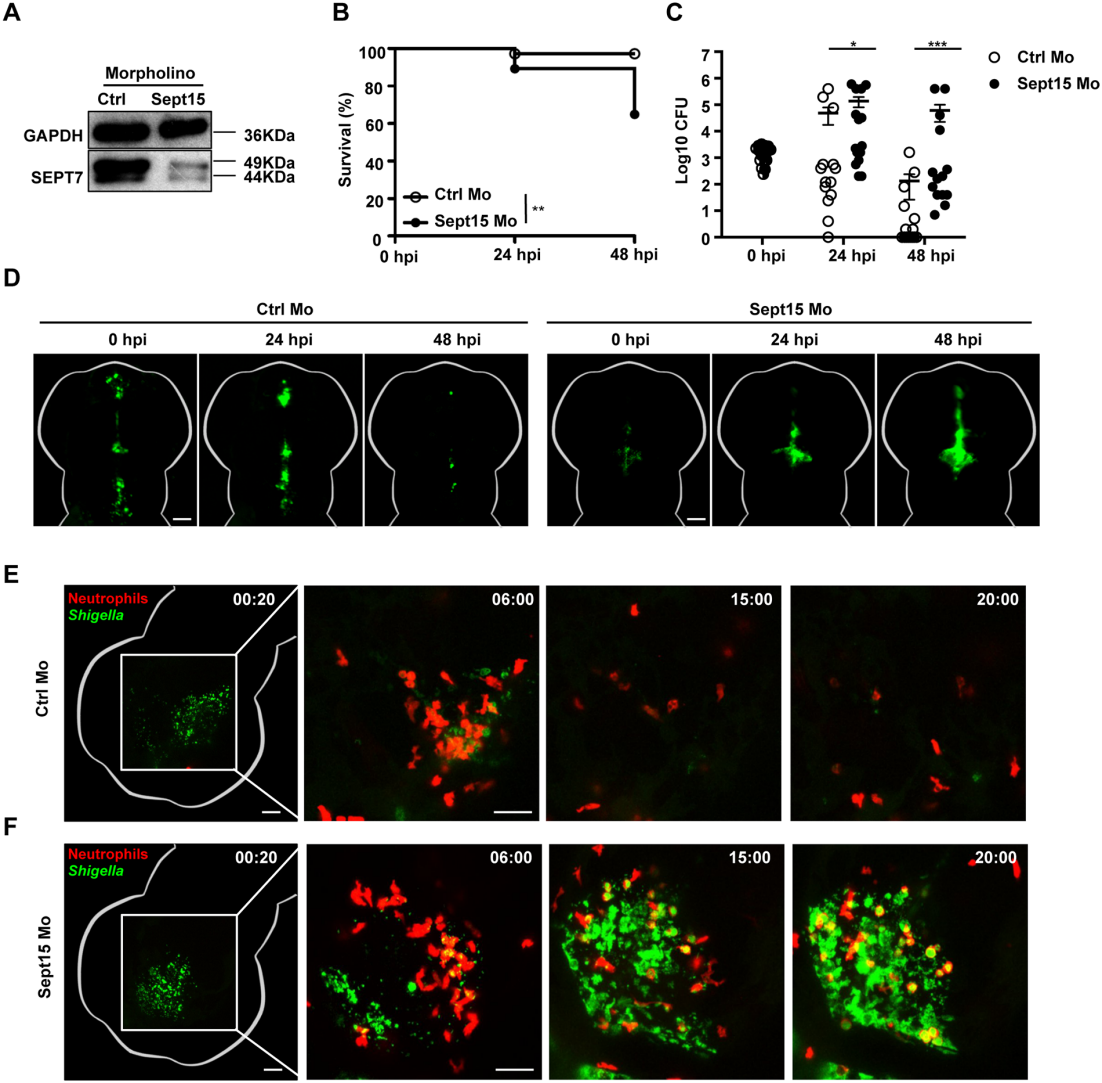
Sept15 morphants show increased susceptibility to *S*. *flexneri* infection. A. Representative western blot of extracts from larvae injected with control (Ctrl) or Sept15 morpholino (Mo) using antibodies against GAPDH (as control) or SEPT7. B. Survival curves of Ctrl or Sept15 morphants infected with *S*. *flexneri* M90T (low dose). Pooled data from 3 independent experiments per treatment using at least 15 larvae per treatment. Significance testing performed by Log Rank test. **, P<0.01. C. Enumeration of bacteria at 0, 24, or 48 hpi from Ctrl (open circles) or Sept15 (closed circles) morphants infected with *S*. *flexneri* M90T (low dose). Half-filled circles represent enumerations from larvae at time 0 and are representative of inoculums for both conditions. Pooled data from 3 independent experiments using up to 3 larvae per treatment. Circles represent individual larvae, and only larvae having survived the infection (thus far) included here (i.e., dead larvae not homogenised for counts). Mean ± SEM also shown (horizontal bars). Significance testing performed by Student’s t test. *, P<0.05; ***, P<0.001. D. Representative images of larvae injected with Ctrl or Sept15 Mo and infected in the HBV with GFP-*S*. *flexneri* M90T (low dose). For each treatment, the same larva was imaged at 0, 24, and 48 hpi using a fluorescent stereomicroscope. Scale bars, 100 μm. See also S4 and S5 Videos. E-F. Representative frames extracted from *in vivo* time-lapse confocal imaging of *lyz*:dsRed larvae (3 dpf, red neutrophils) injected with (E) Ctrl or (F) Sept15 Mo and infected in the HBV with GFP-*S*. *flexneri* M90T (low dose). First frame 20 mpi, followed by frames at 6, 15, and 20 hpi. Maximum intensity Z-projection images (2 μm serial optical sections) are shown. Scale bars, 50 μm. See also S6 and S7 Videos.

Structural analysis of a human septin complex revealed that SEPT7 is essential for septin filament assembly and function [25]. Zebrafish have orthologs for members of all 4 human septin groups (S1 Table), including Sept15 and Sept7b which share 88.7% and 92.5% identity with human SEPT7, respectively [26]. Confocal microscopy of zebrafish larvae labeled with human anti-SEPT7 antibody shows that Sept15 and/or Sept7b are present in epithelial cells, macrophages, and neutrophils (S2A Fig). To investigate the role of septins in host defence *in vivo*, zebrafish larvae were injected with control or Sept15 morpholino oligonucleotide and infected with *S*. *flexneri* (Fig 2A). As compared to infected control morphants, infected Sept15 morphants present significantly reduced survival and higher bacterial loads (Fig 2B and 2C). Using fluorescent microscopy we observed that in the absence of Sept15 zebrafish larvae failed to clear the infection, showing increasing fluorescence of GFP-*Shigella* over time (Fig 2D, see also S4 and S5 Videos). In contrast to the ~40% mortality observed for Sept15 morphants infected with wild type *S*. *flexneri*, Sept15 morphants infected with avirulent T3SS- *S*. *flexneri* present ~100% survival (S2B and S2C Fig). Together, these results show that susceptibility of Sept15 morphants to *S*. *flexneri* infection is dependent on the T3SS, and suggest that septins have an important role in the control of *S*. *flexneri* infection *in vivo*. To test if these results are specific to Sept15, we performed experiments using a morpholino oligonucleotide against Sept7b (S2D Fig). Similar to results obtained for Sept15 morphants, Sept7b morphants infected with *S*. *flexneri* present significantly reduced survival and higher bacterial loads as compared to infected control morphants (S2E and S2F Fig). To test if the impact of septin depletion is specific to infection of the HBV, Sept15 morphants were systemically infected with *S*. *flexneri* via the caudal vein (S2G and S2H Fig). In the case of caudal vein infection, Sept15 morphants present ~40% mortality as compared to control morphants which showed 100% survival.

Neutrophils are crucial to control *Shigella* infection *in vivo* [6]. To characterize the ability of Sept15-depleted neutrophils to clear GFP-*S*. *flexneri*, we analyzed *S*. *flexneri*-neutrophil interactions at the level of the single cell using high-resolution confocal microscopy (Fig 2E and 2F, see also S6 and S7 Videos). In both control and Sept15 morphants, neutrophils are massively recruited to the infection site where they engulf bacteria. Time-lapse movies confirmed that neutrophils from control morphants reliably clear a low dose of GFP-*Shigella*. In contrast, neutrophils from Sept15 morphants are unable to clear the same dose of *Shigella*, and are killed upon bacterial challenge.

### *S*. *flexneri* induces neutrophil death in Sept15 morphants

**Fig 3:**
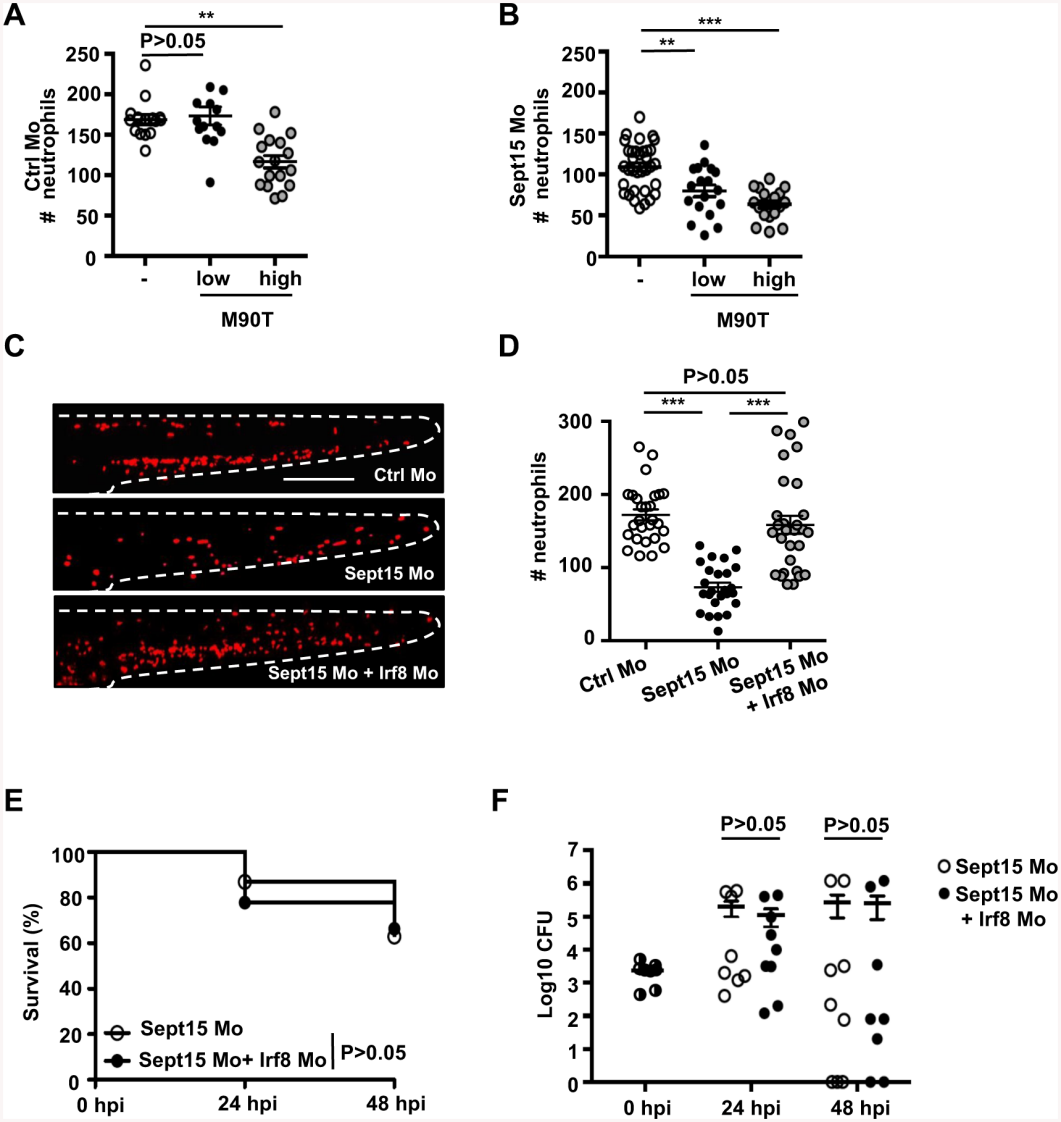
*S*. *flexneri* induces neutrophil death in Sept15 morphants. A-B. Quantification of neutrophils in *lyz*:dsRed larvae injected with (A) Ctrl or (B) Sept15 morpholino (Mo), uninfected (open circles) or infected for 6 h with a low (closed circles) or high (grey circles) dose of *S*. *flexneri* M90T, from 4 or more larvae per treatment from 3 independent experiments. Circles represent individual larvae. Mean ± SEM also shown (horizontal bars). Significance testing performed by ANOVA with Bonferroni posttest. **, P<0.01; ***, P<0.001. C. Representative images of *lyz*:dsRed larvae injected with Ctrl, Sept15, or Sept15 + Irf8 Mo. Scale bar, 250 μm. D. Quantification of neutrophils in *lyz*:dsRed larvae injected with Ctrl (open circles), Sept15 (closed circles), or Sept15 + Irf8 (grey circles) Mo. Circles represent individual larvae. Mean ± SEM also shown (horizontal bars). Significance testing performed by ANOVA with Bonferroni posttest. ***, P<0.001. E. Survival curves of Sept15 or Sept15 + Irf8 morphants infected with *S*. *flexneri* M90T (low dose). Pooled data from 3 independent experiments per treatment using at least 15 larvae per treatment. Significance testing performed by Log Rank test. F. Enumeration of bacteria at 0, 24, or 48 hpi from Sept15 (open circles) or Sept15 + Irf8 (closed circles) morphants infected with *S*. *flexneri* M90T (low dose). Half-filled circles represent enumerations from larvae at time 0 and are representative of inocula for both conditions. Pooled data from 3 independent experiments per inoculum class using up to 3 larvae per treatment. Circles represent individual larvae, and only larvae having survived the infection (thus far) included here (i.e., dead larvae not homogenised for counts). Mean ± SEM also shown (horizontal bars). Significance testing performed by Student’s t test.

To investigate the fate of neutrophils during infection of Sept15-depleted zebrafish, we used live cell imaging and monitored neutrophils in *lyz*:dsRed control or Sept15 morphants infected with GFP-*S*. *flexneri*. We quantified the total number of neutrophils at the whole animal level in control or Sept15 morphants at 3 dpf. Whereas neutrophil numbers in control morphants infected for 6 h with a low dose of *S*. *flexneri* are not significantly different from PBS-injected larvae, larvae infected for 6 h with a high dose of *S*. *flexneri* are neutropenic (Fig 3A). Sept15 morphants develop fewer neutrophils than control morphants, and when infected for 6 h with a low or high dose of *S*. *flexneri*, neutrophils were reduced even further (Fig 3B). The infection-mediated decrease in neutrophils is dependent on the T3SS, as infection with T3SS- *S*. *flexneri* has no effect on neutrophil number in either control or Sept15 morphants (S3A and S3B Fig). To test if increased mortality in Sept15 morphants is a result of neutropenia, we co-injected control or Sept15 morphants with a morpholino oligonucleotide against Irf8 (a gene involved in leukocyte differentiation [27]) to skew the myeloid cell balance towards neutrophils, and infected larvae with *S*. *flexneri* (Fig 3C and 3D). Increasing the number of neutrophils is unable to rescue Sept15 morphants from mortality or increasing bacterial burdens, suggesting that susceptibility of Sept15 morphants to *S*. *flexneri* is not because of a reduction in neutrophils *per se* (Fig 3E and 3F). Depletion of macrophages by Irf8 knockdown [27] may also contribute to the susceptibility of Sept15 morphants. Indeed, the ablation of macrophages by exposure of the transgenic line Tg(*mpeg1*:G/U:mCherry) to metronidazole showed that macrophages provide some protection against high dose *S*. *flexneri* infection (S3C and S3D Fig), likely because macrophages are implicated in the initial phagocytosis of *Shigella* and facilitate neutrophil scavenging crucial for host defence [6].

### Sept15 restricts the inflammatory response *in vivo*

**Fig 4:**
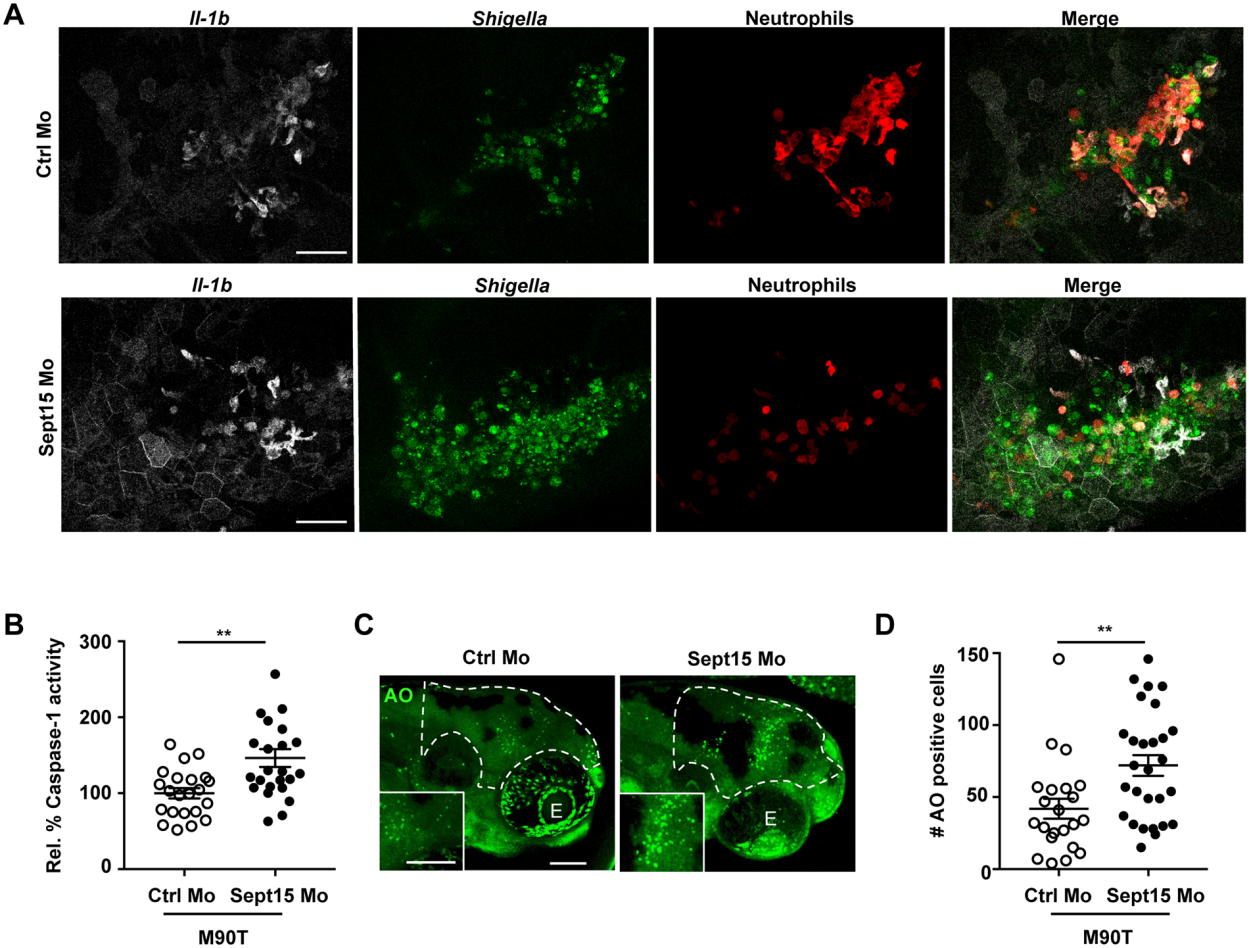
Sept15 restricts the inflammatory response *in vivo*. A. Representative frames extracted from *in vivo* time-lapse confocal imaging of Tg(*il-1b*:GFP-F) x Tg(*lyz*:dsRed) larvae (3 dpf) injected with Ctrl or Sept15 Mo and infected in the HBV with Crimson-*S*. *flexneri* M90T (low dose) at 19 hpi. Maximum intensity Z-projection images (2 μm serial optical sections) are shown. Scale bars, 50 μm. See also S8 and S9 Videos. B. Relative % of Caspase-1 activity levels measured in Ctrl and Sept15 morphants infected for 6 h with ~5 x 10^3^ CFU of *S*. *flexneri* M90T. Mean±SEM from 3 independent experiments per treatment using 5–10 larvae per experimental group. Significance testing performed by Student’s t test. **; P<0.01. C. Representative images of Ctrl and Sept15 morphants (Mo) infected with *S*. *flexneri* M90T in the HBV and stained for acridine orange. Dotted line shows the area where cells were quantified (i.e. the infected HBV). Scale bars, 100 μm. E, eye. D. Number of acridine orange (AO) positive cells counted in the HBV of Ctrl or Sept15 morphants 6 hpi with *S*. *flexneri* M90T. Significance testing performed by Student’s t test. **, P<0.001. Pooled data from 3 independent experiments per treatment using at least 6 larvae per treatment.

*S*. *flexneri* is well known to induce inflammation *in vitro* and *in vivo* [6, 28, 29]. To identify sources of inflammation in septin-depleted zebrafish larvae, we followed the spatio-temporal dynamics of interleukin 1 beta (*il-1b*) induction during *S*. *flexneri* infection. For this we outcrossed Tg(*il-1b*:GFP-F)^*zf550*^ (herein referred to as *il-1b*:GFP-F) a transgenic line which expresses farnesylated GFP under control of the *il-1b* promoter [30], with *lyz*:dsRed for live cell analysis by confocal microscopy (Fig 4A, see also S8 and S9 Videos). In both control and Sept15 morphants infected with Crimson-*S*. *flexneri*, we observed *il-1b*:GFP-F expression in neutrophils, macrophages, and epithelial cells surrounding the infection site, indicating that leukocytes and other cell types can be a source of *il-1b* during *S*. *flexneri* infection. To understand why Sept15 morphants succumb to *S*. *flexneri* infection, we tested Caspase-1 activity (as a readout of Il-1β processing and maturation [31]) in *Shigella*-infected larvae using FAM-YVAD-FMK, a fluorochrome-labeled inhibitor of Caspase-1 (FLICA) that binds specifically to active Caspase-1 enzyme. Strikingly, Caspase-1 activity is significantly increased in Sept15 morphants compared to control morphants (Fig 4B). Caspase-1-mediated signaling pathways are closely-linked to host cell death [32]. To test whether increased mortality in Sept15-depleted larvae correlates with increased host cell death, we quantified dying cells in the HBV of control and Sept15 morphants infected for 6 h with *S*. *flexneri* using acridine orange (AO), a nucleic acid-binding dye which marks dying cells (Fig 4C and 4D). In agreement with increased Caspase-1 activity, we detected a significant increase in numbers of AO-positive cells (1.7 ± 0.3 fold) in *S*. *flexneri*-infected Sept15 morphants as compared to control morphants. Together, these results suggest that hyper-inflammation is an underlying factor in the susceptibility of Sept15-deficient larvae to *S*. *flexneri* infection.

Previous work has shown that overexpression of leukotriene A_4_ hydrolase (*lta4h*) generates inflammation due to induction of tumor necrosis factor alpha (*tnf-a*), making zebrafish more susceptible to infection by *Mycobacterium marinum* [33]. To distinguish between *tnf-a* and *il-1b* inflammatory pathways in host defence against *S*. *flexneri*, we overexpressed *lta4h* in zebrafish larvae (S4 Fig). Overexpression of *lta4h* significantly increased transcript levels of *tnf-a* without affecting levels of *il-1b* (S4A and S4B Fig). However, the upregulation of *tnf-a* failed to increase susceptibility to a low dose of *S*. *flexneri* infection (S4C and S4D Fig), strongly suggesting that increased susceptibility of Sept15 morphants to *S*. *flexneri* infection is dependent on the activation of an *il-1b* signaling cascade.

### Reduction of inflammation by anakinra rescues Sept15-deficiency *in vivo*

**Fig 5:**
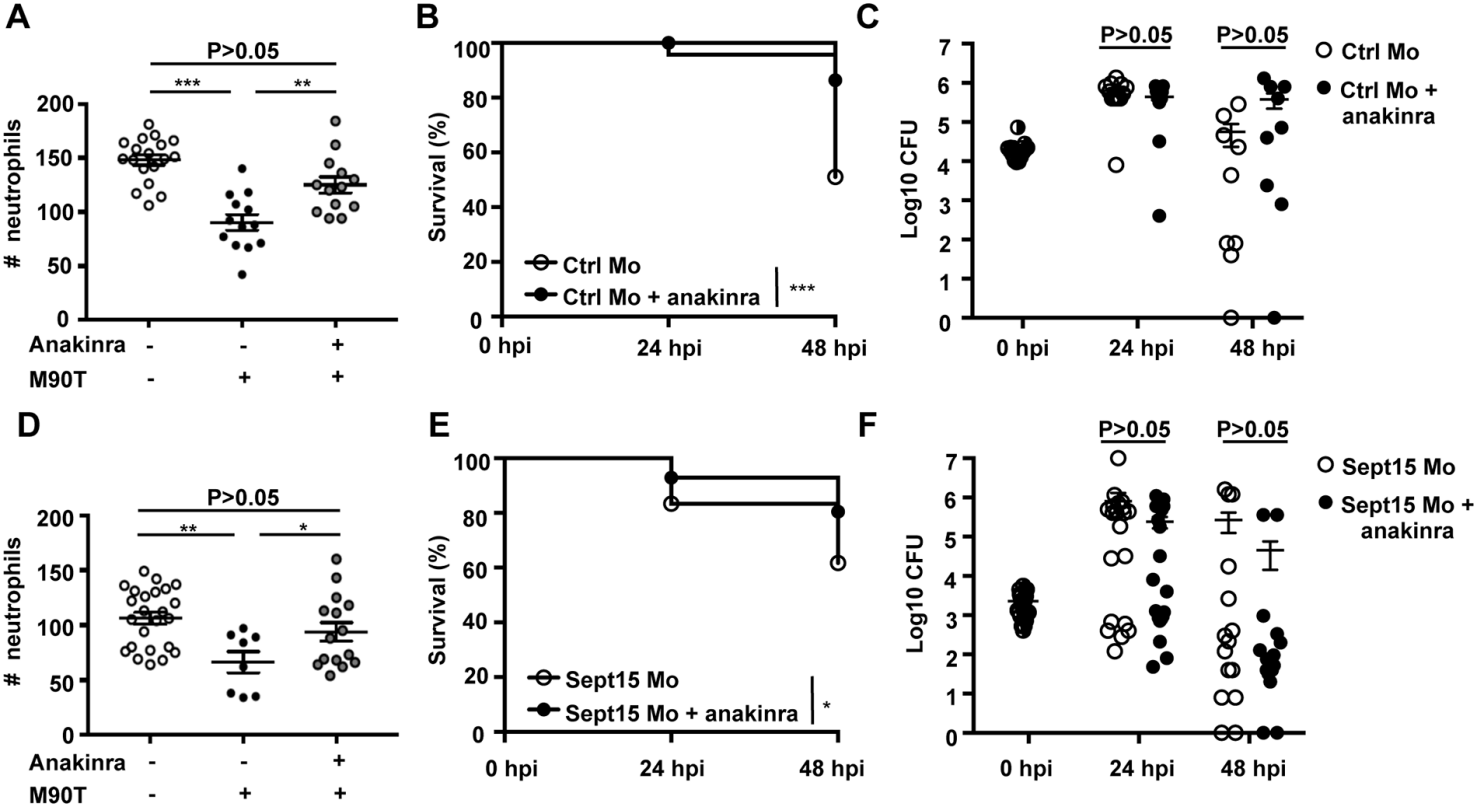
Anakinra therapy protects neutrophils and enhances host survival upon *S*. *flexneri* infection. A. Quantification of neutrophils in *lyz*:dsRed larvae injected with Ctrl morpholino, uninfected (open circles) or infected for 6 h with a high dose of *S*. *flexneri* M90T (closed circles) and treated with anakinra (grey circles), from 3 independent experiments using up to 5 larvae per treatment. Circles represent individual larvae. Mean ± SEM also shown (horizontal bars). Significance testing performed by ANOVA with Bonferroni posttest. ***, P<0.001. B. Survival curves of Ctrl morphants infected with *S*. *flexneri* M90T (high dose), untreated (open circles) or treated with anakinra (closed circles). Pooled data from 4 independent experiments per treatment using at least 15 larvae per experiment. Significance testing performed by Log Rank test. **, P<0.01. C. Enumeration of bacteria at 0, 24, or 48 hpi from Ctrl morphants infected with *S*. *flexneri* M90T (high dose), untreated (open circles) or treated with anakinra (closed circles). Half-filled circles represent enumerations from larvae at time 0 and are representative of inoculums for both conditions. Pooled data from 4 independent experiments per inoculum class using up to 3 larvae per treatment. Circles represent individual larvae, and only larvae having survived the infection (thus far) included here (i.e., dead larvae not homogenised for counts). Mean ± SEM also shown (horizontal bars). Significance testing performed by Student’s t test. D. Quantification of neutrophils in *lyz*:dsRed larvae injected with Sept15 Mo, uninfected (open circles) or infected for 6 h with a low dose of *S*. *flexneri* M90T (closed circles) and treated with anakinra (grey circles), from 3 independent experiments using up to 3 larvae per treatment. Circles represent individual larvae. Mean ± SEM also shown (horizontal bars). Significance testing performed by ANOVA with Bonferroni posttest. **, P<0.01. E. Survival curves of Sept15 morphants infected with *S*. *flexneri* M90T (low dose), untreated (open circles) or treated with anakinra (closed circles). Pooled data from 5 independent experiments per treatment using at least 15 larvae per experiment. Significance testing performed by Log Rank test. **, P<0.01. F. Enumeration of bacteria at 0, 24, or 48 hpi from Sept15 morphants infected with *S*. *flexneri* M90T (low dose), untreated (open circles) or treated with anakinra (closed circles). Half-filled circles represent enumerations from larvae at time 0 and are representative of inoculums for both conditions. Pooled data from 7 independent experiments per treatment using up to 3 larvae per experiment. Circles represent individual larvae, and only larvae having survived the infection (thus far) included here (i.e., dead larvae not homogenised for counts). Mean ± SEM also shown (horizontal bars). Significance testing performed by Student’s t test.

Anakinra is an antagonist of IL-1 receptor (IL-1R) used to prevent inflammatory shock, sepsis, and auto-inflammatory syndromes in humans [34, 35]. Anakinra is an analogue of human IL-1RA (interleukin 1 receptor antagonist), an endogenous inhibitor of IL-1 that binds competitively to the IL-1 receptor. Although an endogenous Il-1β receptor antagonist has not been reported in fish, anakinra presents comparable homology to both human and zebrafish IL-1β (31.0% and 29.7% respectively). We tested the ability of anakinra to reduce inflammation and increase protection in our *S*. *flexneri*-zebrafish infection model. Treatment with anakinra rescued the survival of neutrophils and larvae infected with a high dose of *S*. *flexneri* (Fig 5A and 5B), without significantly affecting the enumerations of bacterial burden quantified from viable larvae (Fig 5C).

We next tested the protective effect of anakinra in Sept15 morphants. In the absence of infection, neutrophil numbers do not differ between control and anakinra-treated morphants (S5A Fig). Remarkably, upon *Shigella* infection, anakinra prevented neutrophil death and significantly reduced the mortality of infected Sept15 morphants (Fig 5D and 5E), without significantly affecting enumerations of bacterial burden quantified from viable larvae (Fig 5F). Collectively, these results show that reduction of inflammation by therapy can promote neutrophil and zebrafish survival during *S*. *flexneri* infection, and rescue septin-deficiency *in vivo*.

## Discussion

The zebrafish is a powerful non-mammalian vertebrate model to study the innate immune response to bacterial infection [36, 37]. We have previously used *Shigella* infection of the zebrafish caudal vein to study bacterial autophagy *in vivo* [6]. Here, using *Shigella* infection of the zebrafish HBV, we reveal that septins have a crucial role in restricting inflammation *in vivo*. Strikingly, anakinra is able to counteract septin-deficiency by preventing neutrophil death and reducing zebrafish mortality upon *S*. *flexneri* infection. These findings reveal a novel role for septins in inflammation control and host defence.

The zebrafish HBV has been used to model infection by other bacterial pathogens including *Listeria monocytogenes* [38], *Salmonella* Typhimurium [39], *Pseudomonas aeruginosa* [40, 41], and *M*. *marinum* [42]. In the case of *L*. *monocytogenes*, bacteria in the HBV disseminate 2–3 dpi, spreading infection to the trunk and tail muscle. Although *S*. *flexneri* is well known for invasion and inflammatory destruction of the human intestinal epithelium, and similarly to *L*. *monocytogenes* has the ability to form actin tails and spread from cell-to-cell [43], we did not observe *S*. *flexneri* dissemination outside of the zebrafish HBV or forebrain ventricle. This allowed us to analyze *S*. *flexneri*-neutrophil interactions in a compartmentalized environment, where we observed that recruited neutrophils efficiently engulf and eliminate a low dose of S. *flexneri*. This neutrophil behavior is in stark contrast to HBV infections of non-pathogenic *E*. *coli*, where neutrophils poorly engulf fluid-borne bacteria [44]. These observations are likely a result of *S*. *flexneri* virulence factors which promote bacterial recognition and engulfment by neutrophils.

The rabbit ileal loop model is commonly used to study the host response to *Shigella* infection [28]. Recently, a mouse model of shigellosis by intraperitoneal infection has been described [29]. In both animal models, *S*. *flexneri* induces the expression of proinflammatory cytokines, including IL-1β and TNF-α, as observed in humans suffering from shigellosis [45]. However, mammalian models remain poorly suited to spatio-temporally examine the innate immune response to *Shigella in vivo*. By contrast, the natural translucency of zebrafish larvae enables non-invasive *in vivo* imaging at high resolution throughout the organism. We show that *il-1b*:GFP-F larvae can be used to visualize the spatio-temporal dynamics of *il-1b* during *S*. *flexneri* infection. In-depth investigation of infection by *Shigella* and other bacteria that induce inflammatory signals, including *L*. *monocytogenes* and *S*. Typhimurium, will help to describe more precisely the coordination between septin assembly and inflammation.

When applied as a model of vertebrate development, the zebrafish has been key in linking Sept9a and Sept9b to growth defects *in vivo* [46]. In support of a highly conserved role for septins amongst vertebrates, the depletion of Sept15 induces cell differentiation and division defects in the pancreatic endocrine cells of zebrafish larvae [47]. More recently, the zebrafish has been used to highlight the central role of Sept15 in actin-based myofibril and cardiac function [48]. Septins are known components of the ciliary diffusion barrier in humans, and zebrafish Sept6 and Sept15 morphants present phenotypes resembling human ciliopathies, highlighting translatability of the zebrafish as a model for the study of septin biology *in vivo* [26, 49]. Here, we report defects in innate immunity that derive from Sept15 depletion, including inflammation and neutropenia, and show that inflammation increases the susceptibility of neutrophils to *S*. *flexneri* infection. The mechanisms underlying cell death by *Shigella* in epithelial cells [50] and macrophages (including apoptosis [51], necrosis [52], pyroptosis [53], and pyronecrosis [54]), have been the subject of intense investigation. The zebrafish can represent a unique experimental system to investigate *Shigella*-neutrophil interactions and dissect the molecular features underlying *Shigella*-mediated host cell death *in vivo*. Moreover, it is envisioned that insights into neutrophil biology arising from our *S*. *flexneri*-zebrafish model can enable novel therapeutic approaches towards diseases with an important neutrophil component.

The dysregulation of IL-1β is associated with a wide variety of inflammatory diseases [55]. Intervention into this pro-inflammatory pathway, either by blocking IL-1R or by preventing the processing / secretion of IL-1β, is critical for treatment [34]. For example, anakinra has been used to reduce IL-1β levels in a mouse model of chronic granulomatous disease (CGD), an immunodeficiency characterized by defective production of ROS [56]. Anakinra has also been effective in treatment of human patients with Schnitzler syndrome (an autoimmune disorder) or with mutations in cold-induced autoinflammatory syndrome 1 gene (CIAS1) [57]. Results obtained from our *S*. *flexneri*-zebrafish HBV infection model show that septins play a key role in the restriction of inflammation and neutrophil clearance of *S*. *flexneri*. Other studies performed in zebrafish have identified a role for the inflammasome in leukocyte clearance of *L*. *monocytogenes* and *S*. Typhimurium [58, 59]. What is the precise role of septins in inflammation? Septins are a unique component of the cytoskeleton that associate with cellular membranes, actin filaments, and microtubules [1]. Previous work has described a role for the actin cytoskeleton in inflammation control, by regulating the NLRP3 and pyrin inflammasomes [10–14]. We hypothesize that septins interact with components of the inflammasome and regulate assembly of this multiprotein complex. Although a precise role for septins in the assembly and activity of the inflammasome awaits investigation, these results add weight to previous studies linking inflammation and the cytoskeleton, and suggest that targeting the cytoskeleton can represent an important anti-inflammatory strategy. It is increasingly recognized that interactions between inflammation and the cytoskeleton play important roles in determining disease outcome. It will now be of great interest to further study the link between septins and inflammation, and pursue components of the cytoskeleton as novel molecular targets for inhibition of inflammation.

## Material and methods

### Ethics statement

Animal experiments were performed according to the Animals (Scientific Procedures) Act 1986 and approved by the Home Office (Project license: PPL 70/7446).

### Zebrafish care and maintenance

Wild type AB were purchased from the Zebrafish International Resource Center (Eugene, OR). Tg(*mpeg1*:Gal4-FF)^*gl25*^/Tg(*UAS-E1b*:*nfsB*.mCherry)^*c264*^, Tg(*lyz*:dsRed)^*nz50*^, Tg(*mpx*:GFP)^*i114*^, Tg(*mpeg1*:YFP)^*w200*^ and Tg(*il-1b*:GFP-F)^*zf550*^ transgenic zebrafish lines are described previously [24, 30, 60–62]. Eggs were obtained by natural spawning and reared in Petri dishes containing 0.5x E2 water supplemented with 0.3 μg/ml methylene blue (embryo medium) [63]. For microscopy, embryo medium was supplemented with 0.003% 1-phenyl-2-thiourea (Sigma-Aldrich) from 1 dpf to prevent melanization. Both embryos and infected larvae were reared at 28.5°C. All timings in the text refer to the developmental stage at the reference temperature of 28.5°C [64]. Larvae were anesthetized with 200 μg/ml tricaine (Sigma-Aldrich) in embryo medium for injections and during *in vivo* imaging.

### Zebrafish bacterial infections

Bacterial strains used in this study were wildtype invasive *S*. *flexneri* serotype 5a M90T expressing green fluorescent protein (GFP), mCherry, or Crimson (GFP-*S*. *flexneri*, mCherry-*S*. *flexneri*, or Crimson-*S*. *flexneri* respectively) and T3SS− non-invasive variant (*ΔmxiD*) expressing mCherry [6]. *S*. *flexneri* were cultured overnight in trypticase soy broth, diluted 80x in fresh trypticase soy broth, and cultured until A_600nm_ = 0.6. For injection of zebrafish larvae, bacteria were recovered by centrifugation, washed and reconstituted at the desired concentration in PBS with 0.1% phenol red. At 3 dpf, zebrafish larvae were microinjected in the HBV with up to 1 nl bacterial suspension as described previously [65]. A low dose was defined as 0.5–3.0 x 10^3^ CFU; a high dose was defined as 1.0–2.2 x 10^4^ CFU. Inoculums were checked *a posteriori* by injecting into PBS and plating onto Luria Broth (LB) agar. Larvae were maintained in individual wells of 24-well culture dishes containing embryo medium.

### Measurement of bacterial burden

At indicated time points, larvae were sacrificed with tricaine, lysed in PBS with 0.4% Triton X-100 and homogenized. Serial dilutions of homogenates were plated onto LB agar supplemented with the appropriate antibiotic and CFU enumerated after 24 h incubation at 37°C; only fluorescent colonies were scored. Only viable larvae were used for CFU enumerations.

### Morpholino and RNA injections

Antisense morpholino oligonucleotides were obtained from GeneTools (www.gene-tools.com). Morpholino sequence 5’-ACTCACCTTAAACAGGAAAGCAAGC-3’ was designed to target zebrafish Sept15 (ENSDARG00000102889). Morpholino sequence 5’-GAAACATCTTCACTTCGTACCTGAA-3’ was designed to target zebrafish Sept7b (ENSDARG00000052673). A standard morpholino sequence with no known target in the zebrafish genome was used as a control [6]. To increase neutrophil numbers, embryos were injected with Irf8 splice blocking morpholino as previously described [27]. Morpholinos were diluted to the desired final concentrations (0.5 mM for Sept7b and Sept15 morpholinos, 1mM for Irf8 morpholino) in 0.1% phenol red solution (Sigma-Aldrich) and 0.8 nl/embryo injected. For Leukotriene A_4_ hydrolase (*lta4h*) overexpression experiments, 1.2 nl of 200 ng/μl RNA was injected, as previously described [33]. Morpholino and RNA injections were performed on 1–8 cell stage embryos.

### Live imaging, image processing, and analysis

Whole-animal *in vivo* imaging was performed on anaesthetized zebrafish larvae immobilized in 1% low melting point agarose in 60 mm Petri dishes as previously described [65]. Transmission and fluorescence microscopy was done using a Leica M205FA fluorescent stereomicroscope. Imaging was performed with a 10x (NA 0.5) dry objective. Multiple-field Z-stacks were acquired every 15 min for experiments involving neutrophil recruitment to HBV infection. For high resolution confocal microscopy, infected larvae were positioned in 35 mm glass-bottom dishes and immobilized in 1% low melting agarose as described in [65]. Confocal microscopy was performed using Zeiss LSM 710, Leica SPE, or Leica SP8 microscopes and 10x, 20x, 40x oil, or 63x oil immersion objectives. For time-course acquisitions, larvae were maintained at 28.5°C. AVI/MOV files were processed and annotated using ImageJ/FIJI software.

### Histologic sections

Zebrafish larvae were infected with *S*. *flexneri* in the HBV at 3 dpf. At 6 hpi, embryos were fixed with 4% paraformaldehyde overnight at 4°C. Embryos were washed 3 times in PBS and mounted in 1% agarose. The agarose was dehydrated in a series of ethanol from 70 to 100% and then in 100% xylene and embedded in paraffin. Transversal sections of the head were stained with hematoxylin and eosin (H&E). H&E-stained tissues were imaged with an Axio Lab.A1 microscope (Carl Zeiss MicroImaging GmbH, Germany) and images acquired using an Axio Cam ERc5s colour camera. Images were processed using AxioVision (Carl Zeiss MicroImaging GmbH, Germany).

### qRT-PCR

Total RNA from 5 snap-frozen larvae was extracted using RNAqueous Kit (Ambion). cDNA was obtained using QuantiTect reverse transcription kit (Qiagen). Primers for *il-1b* and *tnf-a* were previously described [66]. For each experiment, quantitative PCR was performed in technical duplicate using a Rotor-GeneQ (Qiagen) thermocycler and SYBR green reaction power mix (Applied Biosystems). To normalize cDNA amounts, we used the housekeeping gene *ef1a1l1* [6] and the 2^-ΔΔCT^ method [67].

### Caspase-1 activity and cell death detection

Caspase-1 activity was determined using a FAM-FLICA Caspase-1 Assay Kit (ImmunoChemistry Technologies) as described previously [68]. Briefly, a 150x stock solution of FAM-YVAD-FMK was prepared according to the manufacturer’s guidelines. The stock was diluted to 1x in embryo medium and 5–10 larvae per experimental group bathed in the staining solution from 4.5 hpi to 6 hpi at 28.5°C. For cell death detection, larvae were bathed at 6 hpi in 2 μg/ml Acridine Orange in embryo medium for 30 min. Larvae were washed 3 times in embryo medium prior to imaging by confocal microscopy.

### Whole-mount immunostaining

Wholemount immunostaining of zebrafish was performed using a standard protocol [65]. To detect septins in neutrophils and macrophages, Tg(*mpx*:GFP)^*i114*^ and Tg(*mpeg1*:YFP)^*w200*^ larvae were labelled with human anti-SEPT7 antibody (IBL), respectively.

### Immunoblotting

To extract zebrafish proteins, 5–8 larvae were lysed in lysis buffer (1 M Tris, 5 M NaCl, 0.5 M EDTA, 0.01% Triton X-100) and homogenized with pestles. After centrifugation at 4°C for 15 min, supernatants were run on 8% acrylamide gels. Extracts were blotted with anti-SEPT7 (IBL) or anti-GAPDH (GeneTex) as a loading control.

### Drug treatments

Macrophages were ablated by exposure of Tg(*mpeg1*:Gal4-FF)^*gl25*^/Tg(*UAS-E1b*:*nfsB*.mCherry)^*c264*^ larvae to metronidazole (10 mM, Sigma-Aldrich) in embryo medium supplemented with 1% DMSO (Sigma-Aldrich). Metronidazole was administered at 2 dpf for 24 h and larvae washed 3 times prior to infection. For anakinra experiments, the embryo medium was supplemented with anakinra (10 μM, Kineret) from 1 dpf and refreshed daily until completion of the assay at 48 hpi.

### Statistical analysis

Data are represented as mean ± SEM. Statistical significance was determined using Log Rank test (survival curves), unpaired two-tail Student’s *t* test (on log10 values of CFU counts, and log2 gene expression data), or ANOVA with Bonferroni posttest as specified in the figure legends (neutrophil counts, Caspase-1 activity) using Prism software (GraphPad Software Inc). Data were considered significant when P<0.05 (*), P<0.01 (**), or P<0.001 (***).

## Supporting information

S1 Fig *S*. *flexneri* infection of the zebrafish hindbrain ventricle. A. Transverse section of larvae (3 dpf) infected in the HBV with *S*. *flexneri* M90T (low dose) for 6h. Arrow indicates the localisation of the bacteria inside the ventricle. Scale bar, 50 μm. E, eye; M, midbrain ventricle. B. Survival curves of larvae infected with low (≤ 3 x 10^3^ CFU) or high (≥ 1 x 10^4^ CFU) inoculum of T3SS- *S*. *flexneri (*Δ*mxiD* strain) using at least 15 larvae per experiment. Significance testing performed by Log Rank test. C. Enumeration of bacteria at 0, 24, or 48 hpi from larvae infected with low (open circles) or high (closed circles) dose of *S*. *flexneri* M90T using up to 3 larvae per treatment. Circles represent individual larvae. Mean ± SEM also shown (horizontal bars). Significance testing performed by Student’s t test. **, P<0.01; ***, P<0.001. Note bacterial load does not significantly decrease in highly infected fish because of the high bacteria:leukocyte ratio and thus more time is required to clear the bacterial burden. D. Frames extracted from *in vivo* time-lapse confocal imaging of *mpeg1*:*G/U*:mCherry x *mpx*:GFP larvae (3 dpf) injected in the HBV with low dose of Crimson-*S*. *flexneri*. First frame 20 mpi, followed by frames at 1, 3, and 12 hpi. Maximum intensity Z-projection images (2 μm serial optical sections) are shown. Scale bars, 50 μm. See also S1 Video.
(TIF)

S2 Fig Sept15 and Sept7b morphants show increased susceptibility to *S*. *flexneri* infection. A. Immunostaining of zebrafish larvae at 3 dpf with antibody against SEPT7 (red) in cells of the caudal fin epithelium (a), a neutrophil (*mpx*:GFP labeled) (b), and a macrophage (*mpeg1*:*YFP* labeled) (c). Scale bars, 10 μm. B. Survival curves of Ctrl or Sept15 morphants infected with T3SS- *S*. *flexneri (*Δ*mxiD* strain, low dose). Pooled data from 3 independent experiments per treatment using at least 15 larvae per experiment. Significance testing performed by Log Rank test. C. Enumeration of bacteria at 0, 24, or 48 hpi from Ctrl (open circles) or Sept15 (closed circles) morphants infected with T3SS- *S*. *flexneri* (Δ*mxiD* strain). Circles represent individual larvae, and only larvae having survived the infection (thus far) included here (i.e., dead larvae not homogenised for counts). Half-filled circles represent enumerations from larvae at time 0 and are representative of inoculums for both conditions. Pooled data from 3 independent experiments using up to 3 larvae per treatment. Mean ± SEM also shown (horizontal bars). Significance testing performed by Student’s t test. D. Representative western blot of extracts from larvae injected with Ctrl or Sept7b morpholino (Mo) using antibodies against GAPDH (as control) or SEPT7. E. Survival curves of Ctrl or Sept7b morphants infected in the HBV with *S*. *flexneri* M90T (low dose). Pooled data from 3 independent experiments per treatment using at least 15 larvae per treatment. Significance testing performed by Log Rank test. ***, P<0.001. F. Enumeration of bacteria at 0, 24, or 48 hpi from Ctrl (open circles) or Sept7b (closed circles) morphants infected with *S*. *flexneri* M90T (low dose). Half-filled circles represent enumerations from larvae at time 0 and are representative of inoculums for both conditions. Pooled data from 3 independent experiments using up to 3 larvae per treatment. Circles represent individual larvae, and only larvae having survived the infection (thus far) included here (i.e. dead larvae not homogenised for counts). Mean ± SEM also shown (horizontal bars). Significance testing performed by Student’s t test. *, P<0.05. G. Survival curves of Ctrl or Sept15 morphants infected in the caudal vein with *S*. *flexneri* M90T (low dose). Pooled data from 3 or more independent experiments per treatment using at least 15 larvae per experiment. Significance testing performed by Log Rank test. **, P<0.01. H. Enumeration of bacteria at 0, 24, or 48 hpi from Ctrl (open circles) or Sept15 (closed circles) morphants infected with *S*. *flexneri* M90T in the caudal vein. Circles represent individual larvae, and only larvae having survived the infection (thus far) included here (i.e., dead larvae not homogenised for counts). Half-filled circles represent enumerations from larvae at time 0 and are representative of inoculums for both conditions. Pooled data from 3 independent experiments using up to 3 larvae per treatment. Mean ± SEM also shown (horizontal bars). Significance testing performed by Student’s t test. **, P<0.01.
(TIF)

S3 Fig The role of the T3SS and macrophages in *S*. *flexneri* pathogenesis *in vivo*. A-B. Quantification of neutrophils in *lyz*:dsRed larvae injected with (A) Ctrl or (B) Sept15 morpholino (Mo), uninfected (open circles) or infected for 6 h with a low (closed circles) or high (closed squares) dose of T3SS- *S*. *flexneri (*Δ*mxiD* strain), from 4 or more larvae per treatment from 2 independent experiments. Circles represent individual larvae. Significance testing performed by ANOVA with Bonferroni posttest. C. Survival curves of Ctrl or macrophage ablated (metronidazole treated Tg(*mpeg1*:Gal4-FF)/Tg(*UAS-E1b*:nfsB.mCherry)) larvae infected in the HBV with *S*. *flexneri* M90T (low dose). Pooled data from 3 independent experiments per treatment using at least 15 larvae per treatment. Significance testing performed by Log Rank test. ***, P<0.001. D. Enumeration of bacteria at 0, 24, or 48 hpi from Ctrl (open circles) or macrophage ablated (closed circles) larvae infected with *S*. *flexneri* M90T (low dose). Half-filled circles represent enumerations from larvae at time 0 and are representative of inoculums for both conditions. Pooled data from 3 independent experiments using up to 3 larvae per treatment. Circles represent individual larvae, and only larvae having survived the infection (thus far) included here (i.e., dead larvae not homogenised for counts). Mean ± SEM also shown (horizontal bars). Significance testing performed by Student’s t test.
(TIF)

S4 Fig Upregulation of *tnf-a* does not increase susceptibility to *S*. *flexneri* infection. A-B. Relative expression of *tnf-a* and *il1-b* in larvae injected with *lta4h* RNA (*lta4h* high). Mean ± SEM from 3 independent experiments per treatment using at least 5 larvae per experiment. Significance testing performed by Student’s t test. *, P<0.05. C. Survival curves of control and *lta4h* high larvae infected with *S*. *flexneri* M90T (low dose). Pooled data from 3 independent experiments per treatment using at least 15 larvae per treatment. Significance testing performed by Log Rank test. D. Enumeration of bacteria at 0, 24, or 48 hpi from Ctrl (open circles) or *lta4h* high (closed circles) larvae infected with *S*. *flexneri* M90T (low dose). Half-filled circles represent enumerations from larvae at time 0 and are representative of inocula for both conditions. Pooled data from 3 independent experiments per inoculum class using up to 3 larvae per treatment. Circles represent individual larvae, and only larvae having survived the infection (thus far) included here (i.e., dead larvae not homogenised for counts). Mean ± SEM also shown (horizontal bars). Significance testing performed by Student’s t test.
(TIF)

S5 Fig Anakinra therapy does not restore neutrophil number in Sept15 morphants. A. Quantification of neutrophils in *lyz*:dsRed larvae injected with Sept15 morpholino, untreated (open circles) or treated with anakinra (grey circles), from 3 independent experiments using up to 5 larvae per treatment. Control values also represented in Fig 5D. Circles and squares represent individual larvae. Mean ± SEM also shown (horizontal bars). Significance testing performed by ANOVA with Bonferroni posttest.
(TIF)

S1 Table Classification of zebrafish septins. Zebrafish septins were determined using the Ensembl database (ensembl.org), by searching the zebrafish genome assembly (version GRCz10) for proteins containing the septin-type guanine nucleotide-binding (G) domain (IPR030379). The closest human homologs were identified by BLAST of individual protein sequences to UNIPROT database (www.uniprot.org). When multiple isoforms of zebrafish septins are reported in Ensembl, the one referenced in RefSeq (www.ncbi.nlm.nih.gov/refseq/) is reported here and adopted for protein search. In cases of multiple isoforms referenced in RefSeq, we selected the principal isoform according to annotations reported by Ensembl (APPRIS annotations, http://appris.bioinfo.cnio.es). % identity represents the identity of individual zebrafish septins to the canonical isoform of the closest human septin. Position of the septin-type G-domain was predicted using http://prosite.expasy.org. * partial sequence; ** Incomplete septin-type G-domain.
(TIF)

S1 Video Macrophage and neutrophil interactions with *S*. *flexneri* in the zebrafish hindbrain ventricle (related to S1D Fig). Double transgenic *mpeg1*:*G/U*:mCherry x *mpx*:GFP zebrafish larva infected at 3 dpf in the HBV with a low dose of Crimson-*S*. *flexneri* M90T and live imaged at confocal microscope every 1 min 30 sec from 20 mpi (t = 0 on the movie) until 16 hpi (t = 15 h 45 min on the movie). At the beginning of the acquisition few macrophages (mCherry+ cells) have already been recruited to the injected *S*. *flexneri* (white). Note the macrophage full of bacteria (mCherry+ cell full of white bacteria). At t = 1 h 36 min on the movie, this macrophage rounds up, dies, and is rapidly engulfed by neutrophils (GFP+ cells). Neutrophils are recruited to the injected bacteria from 1 hpi, they accumulate progressively in the HBV interacting with the injected bacteria. Note that the bacteria cluster and aggregate on the walls of the ventricle where they are engulfed by neutrophils. Maximum intensity projection (2 μm Z serial sections) is shown.
(MOV)

S2 Video Neutrophils control *S*. *flexneri* infection in the hindbrain ventricle (related to Fig 1E). *lyz*:dsRed zebrafish larva infected at 3 dpf in the HBV with a low dose of GFP-*S*. *flexneri* M90T and live imaged every 1 min from 20 mpi (t = 0 on the movie) to 20 hpi (t = 19 h 30 min on the movie) using confocal microscopy. Neutrophils are recruited to the bacteria and efficiently engulf them as they cluster and aggregate on the walls of the ventricle. Note that neutrophils act as a swarm in engulfing the bacteria. Maximum intensity projection (2 μm serial Z sections) is shown.
(MOV)

S3 Video Neutrophils succumb to a high dose of GFP-*S*. *flexneri* infection in the hindbrain ventricle (related to Fig 1F). *lyz*:dsRed zebrafish larva infected at 3 dpf in the HBV with a high dose of GFP-*S*. *flexneri* M90T and imaged every 1 min 31 sec from 20 mpi to 20 hpi using confocal microscopy. Neutrophils (dsRed+ cells) are recruited to the bacteria from 1 hpi and engulf them. By 3 h 30 mpi (3 h 00 min on the movie), neutrophils fail to eliminate the engulfed bacteria and start to die, presumably killed by *S*. *flexneri* over time. Note the accumulation of green bacteria inside dsRed+ neutrophils, and eventual death of the larvae. Maximum intensity projection (2 μm serial Z optical sections) is shown.
(MOV)

S4 Video Neutrophils control *S*. *flexneri* in the hindbrain ventricle (related to Fig 2D). *lyz*:dsRed zebrafish embryos were injected with Ctrl morpholino and infected at 3 dpf in the HBV with a low dose of GFP-*S*. *flexneri* M90T, and imaged every 15 min for 45 h using fluorescent stereomicroscopy.
(MOV)

S5 Video Neutrophils in Sept15 morphants fail to control *S*. *flexneri* in the hindbrain ventricle (related to Fig 2D). *lyz*:dsRed zebrafish embryos were injected with Sept15 morpholino and infected at 3 dpf in the HBV with a low dose of GFP-*S*. *flexneri* M90T, and imaged every 15 min for 45 h using fluorescent stereomicroscopy.
(MOV)

S6 Video High resolution imaging of neutrophils controlling *S*. *flexneri* infection (related to Fig 2E). *lyz*:dsRed zebrafish embryos were injected with Ctrl morpholino and infected at 3 dpf in the HBV with a low dose of GFP-*S*. *flexneri* M90T and live imaged every 2 min from 20 mpi (t = 0 on the movie) to 20 h 30 mpi (t = 20 h on the movie) using confocal microscopy. As shown in S2 Video, neutrophils (dsRed+ cells) are recruited to the HBV by 1 hpi where they interact with GFP *S*. *flexneri* clustered and aggregated along the HBV wall. Neutrophils progressively engulf and eliminate *S*. *flexneri*, as well as dying infected macrophages (dsRed- GFP+ cells). Maximum intensity projection (2μm serial Z optical sections) is shown.
(MOV)

S7 Video High resolution imaging of neutrophils in Sept15 morphants failing to control *S*. *flexneri* infection (related to Fig 2F). *lyz*:dsRed zebrafish embryos were injected with Sept15 morpholino and infected at 3 dpf in the HBV with a low dose of GFP-*S*. *flexneri* M90T and imaged every 2 min from 20 mpi (t = 0 on the movie) to 20 h 30 mpi (t = 20 h on the movie) using confocal microscopy. S6 and S7 Videos were acquired at the same time. Note that at the beginning of this movie, neutrophil behaviour from Sept15 morphant is similar to that of Ctrl morphant: neutrophils are recruited by 1 hpi and interact with *S*. *flexneri*, and engulf the bacteria and dying macrophages (dsRed- GFP+ cells). However, by 6 hpi neutrophils fail to control the infection (fail to engulf bacteria, fail to engulf dying macrophages) concomitant with massive proliferation of the bacteria. Maximum intensity projection (2μm serial Z optical sections) is shown.
(MOV)

S8 Video Spatio-temporal induction of *il-1b* in control morphant infected with *S*. *flexneri* (related to Fig 4A). Double transgenic Tg(*il-1b*:GFP-F) x Tg(*lyz*:dsRed) zebrafish embryos injected with Ctrl morpholino and infected at 3 dpf in the HBV with a low dose of Crimson-*S*. *flexneri* M90T (labelled green in the movie) and imaged every 3 min from 20 mpi to 20 h 20 mpi using confocal microscopy. Neutrophil (dsRed+ cells) are recruited to the injected bacteria. *il-1b*:GFP expressing cells (labelled white in the movie) start to appear from 3h 30 mpi. Cells which are *il-1b*+ (membrane) and dsRed- are presumably macrophages. Cells which are *il-1b*+ (membrane) and dsRed+ (cytoplasmic) are presumably neutrophils. At the end of the acquisition bacteria have been engulfed by leukocytes and the infection is controlled. Maximum intensity projection (2μm serial Z optical sections) is shown.
(MOV)

S9 Video Spatio-temporal induction of *il-1b* in Sept15 morphant infected with *S*. *flexneri* (related to Fig 4A). Double transgenic Tg(*il-1b*:GFP-F) x Tg(*lyz*:dsRed) zebrafish embryos were injected with Sept15 morpholino and infected at 3 dpf in the HBV with a low dose of Crimson-*S*. *flexneri* M90T (labelled green in the movie) and imaged every 3 min from 20 mpi to 20 h 20 mpi using confocal microscopy. S8 and S9 Videos were acquired at the same time. Neutrophils (dsRed+ cells) are recruited to the injected bacteria. However Sept15 morphants progressively fail to control *S*. *flexneri*, and neutrophils die concomitant with bacterial proliferation. The first *il-1b* producing cells (here labelled in white) are leukocytes observed from 3 hpi. Strikingly, in contrast to Ctrl morphants, Sept15 morphants show increasing *il-1b* induction in neutrophils, macrophages, and epithelial cells surrounding the infection site. Maximum intensity projection (2μm serial Z optical sections) is shown.
(MOV)
