# Supplementary figures and images for: Septins restrict inflammation and protect zebrafish larvae from Shigella infection
Source: PLoS Pathog. 2017 Jun 26;13(6):e1006467. doi: 10.1371/journal.ppat.1006467 (PMC5507465; doi:10.1371/journal.ppat.1006467)

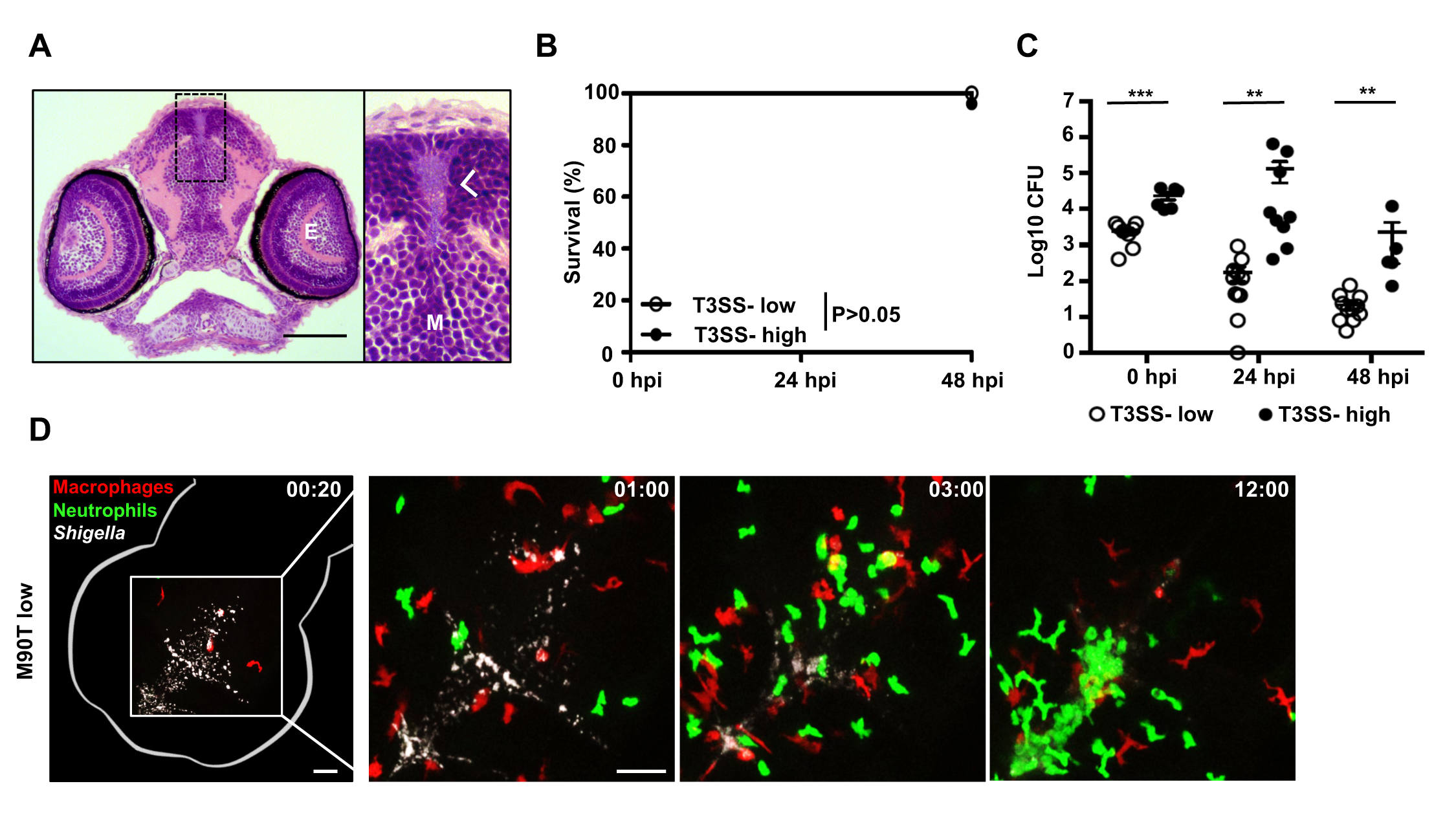

Supplement: S1 Fig — A. Transverse section of larvae (3 dpf) infected in the HBV with S. flexneri M90T (low dose) for 6h. Arrow indicates the localisation of the bacteria inside the ventricle. Scale bar, 50 μm. E, eye; M, midbrain ventricle. B. Survival curves of larvae infected with low (≤ 3 x 10^3^ CFU) or high (≥ 1 x 10^4^ CFU) inoculum of T3SS- S. flexneri (ΔmxiD strain) using at least 15 larvae per experiment. Significance testing performed by Log Rank test. C. Enumeration of bacteria at 0, 24, or 48 hpi from larvae infected with low (open circles) or high (closed circles) dose of S. flexneri M90T using up to 3 larvae per treatment. Circles represent individual larvae. Mean ± SEM also shown (horizontal bars). Significance testing performed by Student’s t test. **, P<0.01; ***, P<0.001. Note bacterial load does not significantly decrease in highly infected fish because of the high bacteria:leukocyte ratio and thus more time is required to clear the bacterial burden. D. Frames extracted from in vivo time-lapse confocal imaging of mpeg1:G/U:mCherry x mpx:GFP larvae (3 dpf) injected in the HBV with low dose of Crimson-S. flexneri. First frame 20 mpi, followed by frames at 1, 3, and 12 hpi. Maximum intensity Z-projection images (2 μm serial optical sections) are shown. Scale bars, 50 μm. See also S1 Video. (TIF) [file ppat.1006467.s001.tif]

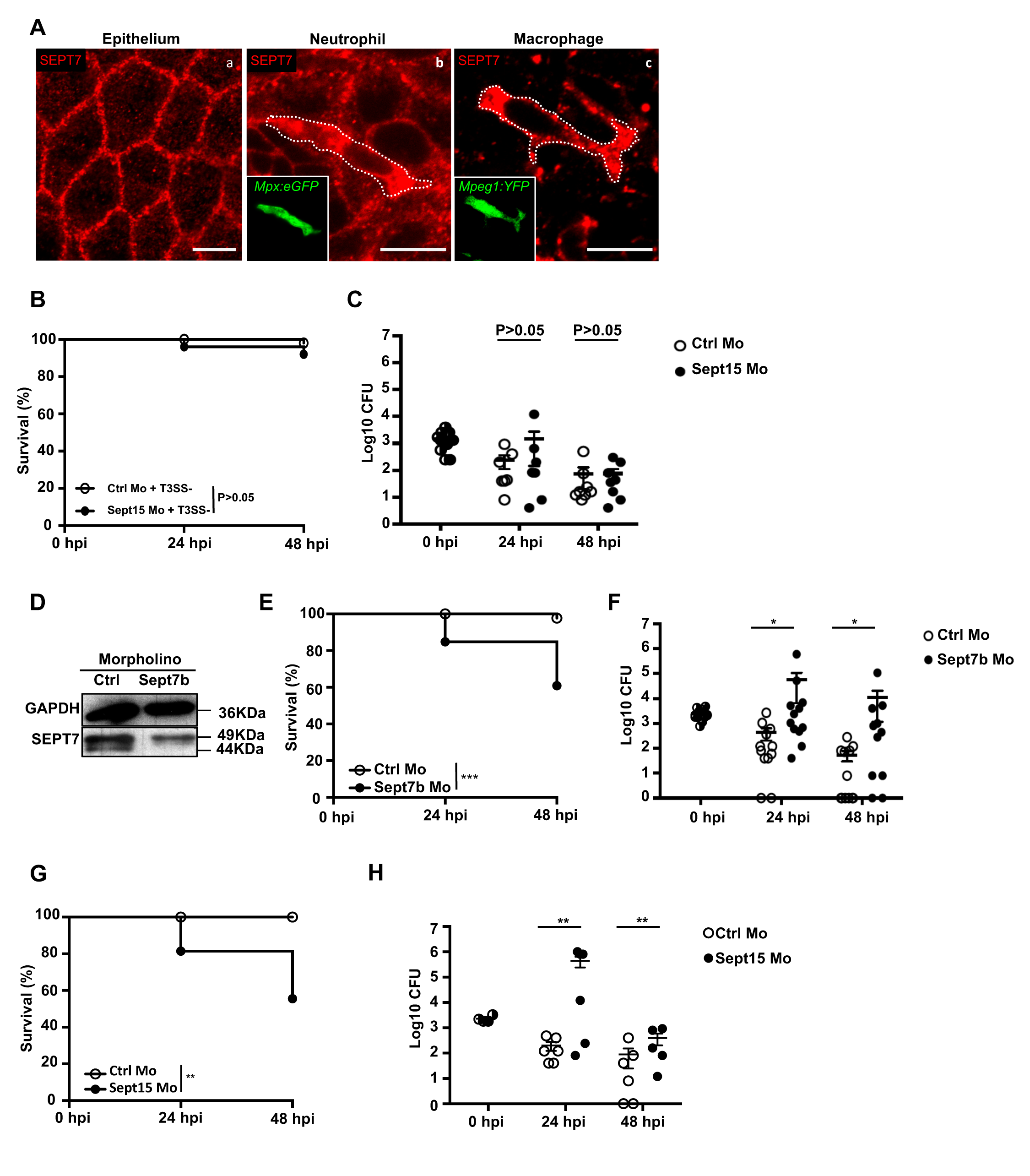

Supplement: S2 Fig — A. Immunostaining of zebrafish larvae at 3 dpf with antibody against SEPT7 (red) in cells of the caudal fin epithelium (a), a neutrophil (mpx:GFP labeled) (b), and a macrophage (mpeg1:YFP labeled) (c). Scale bars, 10 μm. B. Survival curves of Ctrl or Sept15 morphants infected with T3SS- S. flexneri (ΔmxiD strain, low dose). Pooled data from 3 independent experiments per treatment using at least 15 larvae per experiment. Significance testing performed by Log Rank test. C. Enumeration of bacteria at 0, 24, or 48 hpi from Ctrl (open circles) or Sept15 (closed circles) morphants infected with T3SS- S. flexneri (ΔmxiD strain). Circles represent individual larvae, and only larvae having survived the infection (thus far) included here (i.e., dead larvae not homogenised for counts). Half-filled circles represent enumerations from larvae at time 0 and are representative of inoculums for both conditions. Pooled data from 3 independent experiments using up to 3 larvae per treatment. Mean ± SEM also shown (horizontal bars). Significance testing performed by Student’s t test. D. Representative western blot of extracts from larvae injected with Ctrl or Sept7b morpholino (Mo) using antibodies against GAPDH (as control) or SEPT7. E. Survival curves of Ctrl or Sept7b morphants infected in the HBV with S. flexneri M90T (low dose). Pooled data from 3 independent experiments per treatment using at least 15 larvae per treatment. Significance testing performed by Log Rank test. ***, P<0.001. F. Enumeration of bacteria at 0, 24, or 48 hpi from Ctrl (open circles) or Sept7b (closed circles) morphants infected with S. flexneri M90T (low dose). Half-filled circles represent enumerations from larvae at time 0 and are representative of inoculums for both conditions. Pooled data from 3 independent experiments using up to 3 larvae per treatment. Circles represent individual larvae, and only larvae having survived the infection (thus far) included here (i.e. dead larvae not homogenised for counts [file ppat.1006467.s002.tif]

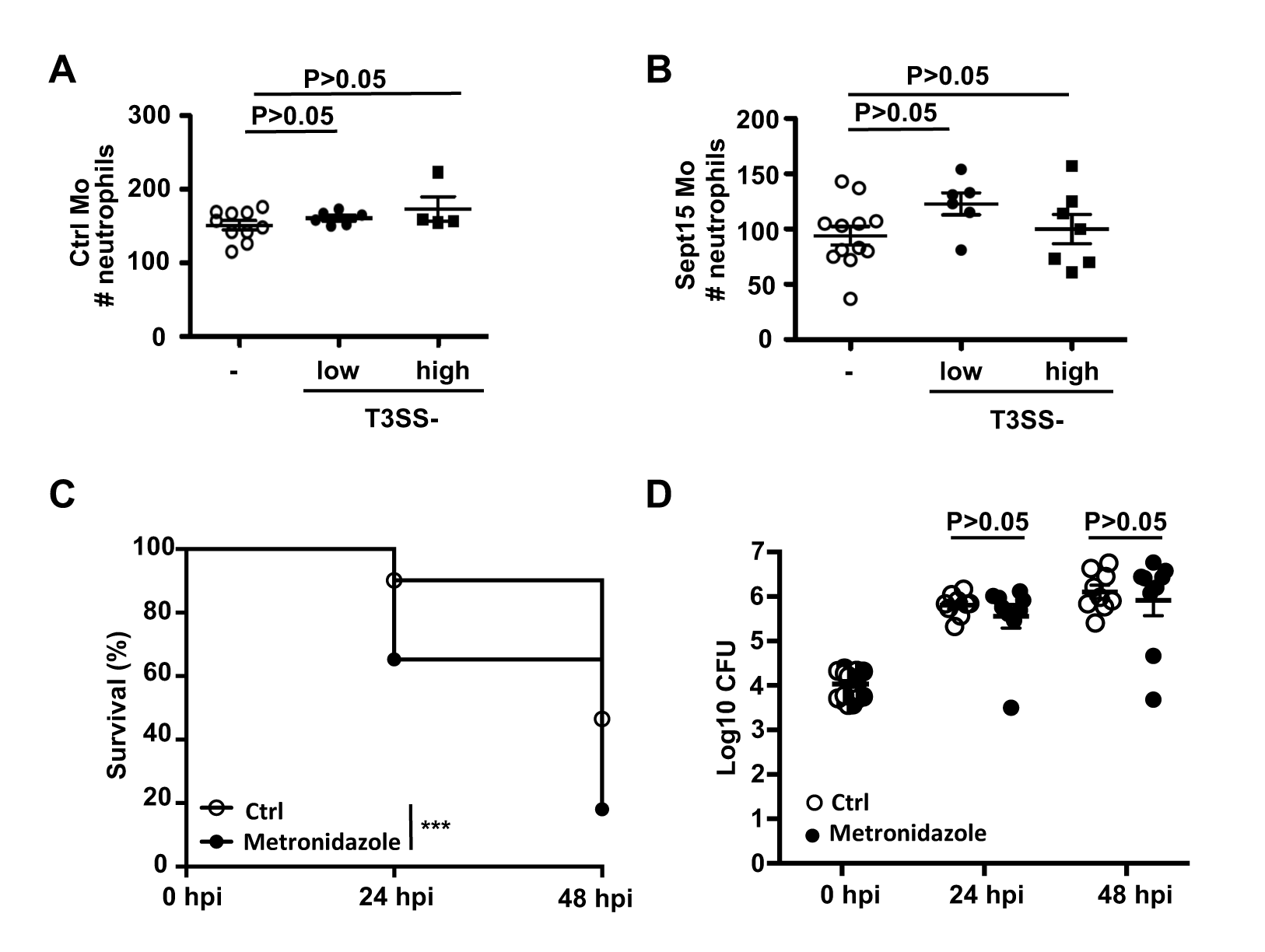

Supplement: S3 Fig — A-B. Quantification of neutrophils in lyz:dsRed larvae injected with (A) Ctrl or (B) Sept15 morpholino (Mo), uninfected (open circles) or infected for 6 h with a low (closed circles) or high (closed squares) dose of T3SS- S. flexneri (ΔmxiD strain), from 4 or more larvae per treatment from 2 independent experiments. Circles represent individual larvae. Significance testing performed by ANOVA with Bonferroni posttest. C. Survival curves of Ctrl or macrophage ablated (metronidazole treated Tg(mpeg1:Gal4-FF)/Tg(UAS-E1b:nfsB.mCherry)) larvae infected in the HBV with S. flexneri M90T (low dose). Pooled data from 3 independent experiments per treatment using at least 15 larvae per treatment. Significance testing performed by Log Rank test. ***, P<0.001. D. Enumeration of bacteria at 0, 24, or 48 hpi from Ctrl (open circles) or macrophage ablated (closed circles) larvae infected with S. flexneri M90T (low dose). Half-filled circles represent enumerations from larvae at time 0 and are representative of inoculums for both conditions. Pooled data from 3 independent experiments using up to 3 larvae per treatment. Circles represent individual larvae, and only larvae having survived the infection (thus far) included here (i.e., dead larvae not homogenised for counts). Mean ± SEM also shown (horizontal bars). Significance testing performed by Student’s t test. (TIF) [file ppat.1006467.s003.tif]

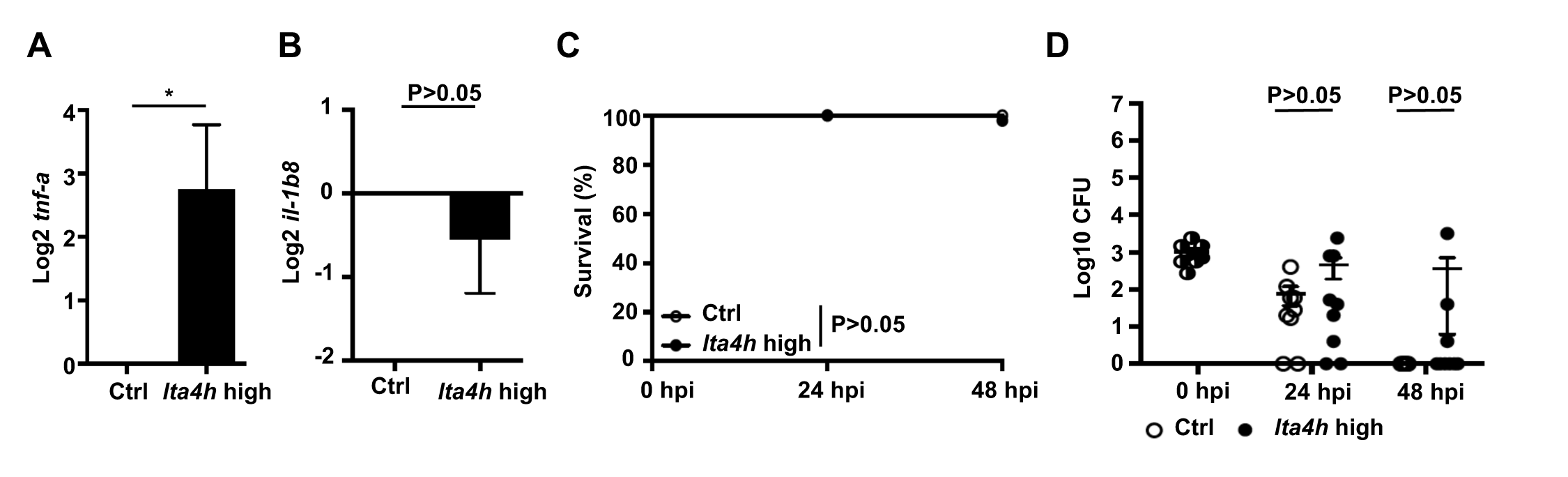

Supplement: S4 Fig — A-B. Relative expression of tnf-a and il1-b in larvae injected with lta4h RNA (lta4h high). Mean ± SEM from 3 independent experiments per treatment using at least 5 larvae per experiment. Significance testing performed by Student’s t test. *, P<0.05. C. Survival curves of control and lta4h high larvae infected with S. flexneri M90T (low dose). Pooled data from 3 independent experiments per treatment using at least 15 larvae per treatment. Significance testing performed by Log Rank test. D. Enumeration of bacteria at 0, 24, or 48 hpi from Ctrl (open circles) or lta4h high (closed circles) larvae infected with S. flexneri M90T (low dose). Half-filled circles represent enumerations from larvae at time 0 and are representative of inocula for both conditions. Pooled data from 3 independent experiments per inoculum class using up to 3 larvae per treatment. Circles represent individual larvae, and only larvae having survived the infection (thus far) included here (i.e., dead larvae not homogenised for counts). Mean ± SEM also shown (horizontal bars). Significance testing performed by Student’s t test. (TIF) [file ppat.1006467.s004.tif]

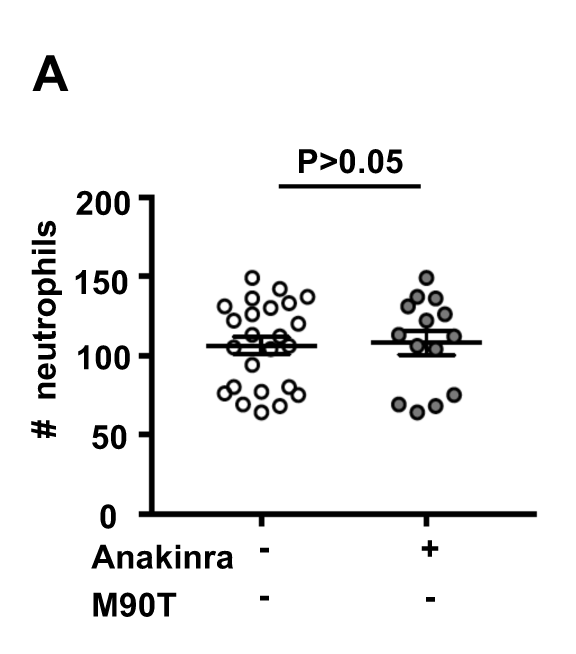

Supplement: S5 Fig — A. Quantification of neutrophils in lyz:dsRed larvae injected with Sept15 morpholino, untreated (open circles) or treated with anakinra (grey circles), from 3 independent experiments using up to 5 larvae per treatment. Control values also represented in Fig 5D. Circles and squares represent individual larvae. Mean ± SEM also shown (horizontal bars). Significance testing performed by ANOVA with Bonferroni posttest. (TIF) [file ppat.1006467.s005.tif]

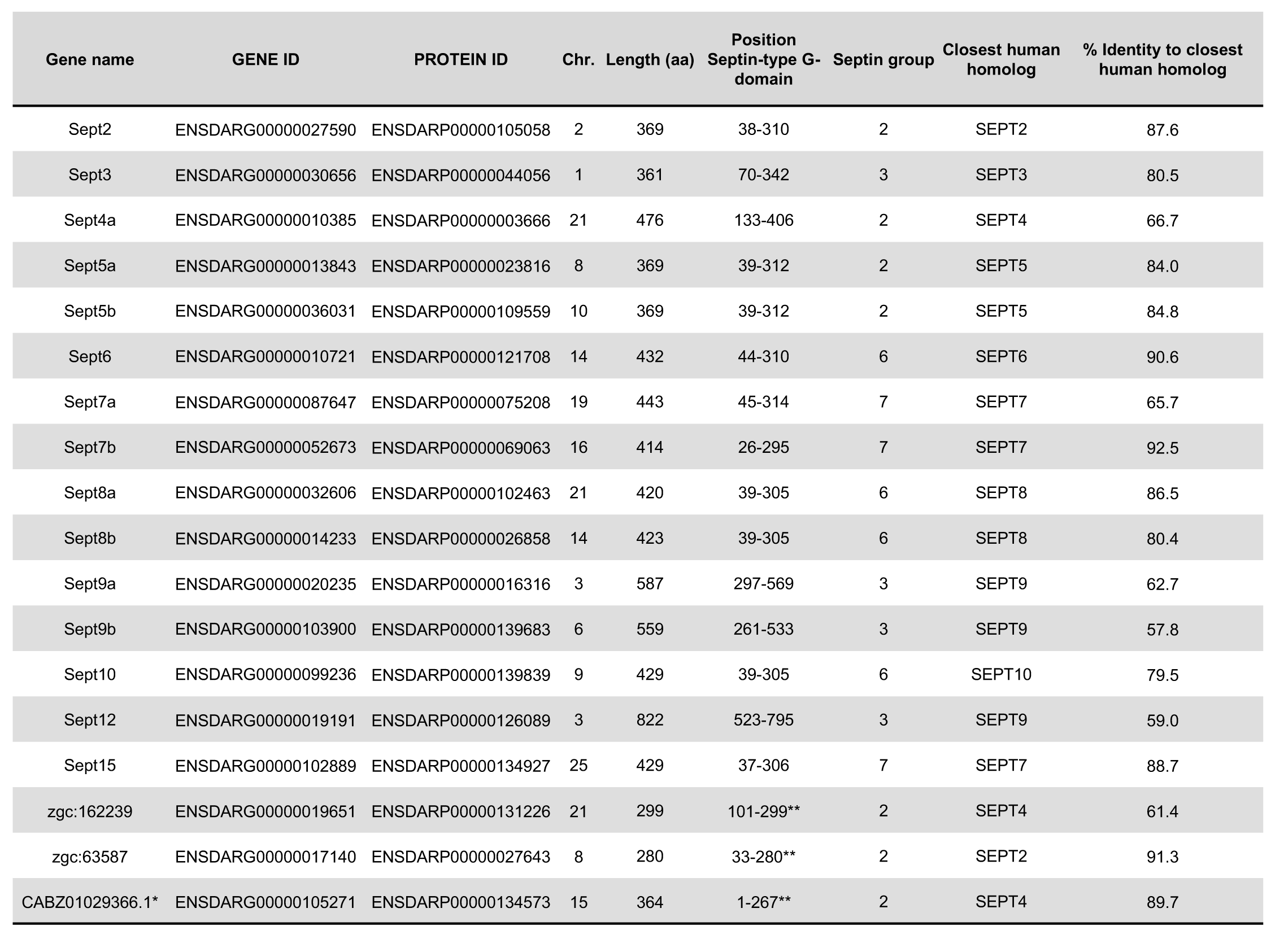

Supplement: S1 Table — Zebrafish septins were determined using the Ensembl database (ensembl.org), by searching the zebrafish genome assembly (version GRCz10) for proteins containing the septin-type guanine nucleotide-binding (G) domain (IPR030379). The closest human homologs were identified by BLAST of individual protein sequences to UNIPROT database (www.uniprot.org). When multiple isoforms of zebrafish septins are reported in Ensembl, the one referenced in RefSeq (www.ncbi.nlm.nih.gov/refseq/) is reported here and adopted for protein search. In cases of multiple isoforms referenced in RefSeq, we selected the principal isoform according to annotations reported by Ensembl (APPRIS annotations, http://appris.bioinfo.cnio.es). % identity represents the identity of individual zebrafish septins to the canonical isoform of the closest human septin. Position of the septin-type G-domain was predicted using http://prosite.expasy.org. * partial sequence; ** Incomplete septin-type G-domain. (TIF) [file ppat.1006467.s006.tif]
